# The burden of Burkitt lymphoma in Africa

**DOI:** 10.1186/s13027-019-0236-7

**Published:** 2019-08-01

**Authors:** Lucia Hämmerl, Murielle Colombet, Rosemary Rochford, David Martin Ogwang, Donald Maxwell Parkin

**Affiliations:** 1 Institute of Medical Epidemiology, Biometrics and Informatics, Medical Faculty of Martin Luther University Halle-Wittenberg Germany, Magdeburger Straße 8, 06112 Halle, Germany; 2 Section of Cancer Information, International Agency for Research on Cancer, 150 cours Albert Thomas, 69372 Lyon Cedex 08, France; 3 Department of Immunology and Microbiology, University of Colorado Anschutz Medical Campus, Aurora, CO USA; 4 Gulu Cancer Registry, St. Mary’s Hospital Lacor, P.O. Box 180, Gulu, Uganda; 5 Nuffield Department of Population Health, University of Oxford, Oxford, OX3 7FL UK; 6 African Cancer Registry Network, 267 Banbury Road, Oxford, OX2 7HT UK

**Keywords:** Burkitt lymphoma, Africa, Epstein Barr virus, Epidemiology, Incidence

## Abstract

**Background:**

Burkitt lymphoma (BL) is a relatively common cancer of childhood in tropical Africa, although its precise incidence and continent-wide geographic distribution have not been previously systematically studied.

**Methods:**

Using the methods employed to produce national estimates of cancer incidence for the “Globocan” series of the International Agency for Research on Cancer, along with detailed information on cancer incidence by histological subtype from cancer registries in Africa, we estimate the numbers and rates of incidence by sex, age group, country and region of Africa.

**Results:**

We estimate that the number of new cases that occurred in 2018 to be about 3900, two thirds in males, and 81% in children aged 0–14. On a national basis, the geographic distribution of incidence rates among children in sub-Saharan Africa resembles that of the prevalence of infection with Falciparum malaria. An estimated 81% of cases are associated with infection with Epstein Barr virus (EBV).

**Conclusions:**

BL comprises almost 50% of childhood of non-Hodgkin lymphoma in Africa, almost all of which are associated with EBV, with the geographic distribution – at least in sub Saharan Africa - mediated by infection with malaria.

## Background

Burkitt lymphoma (BL), an aggressive B cell lymphoma first recognized as a tumour of African children, occurs throughout the world, but has a markedly different incidence in different world regions, and even within regions [1]. By far the highest incidence rates of BL are found in tropical African countries, where it may account for up to half of all childhood cancers [2], and the tumour in these regions is consequently referred to as “endemic BL.” Other African countries outside the equatorial belt have much lower incidence rates, which are similar to those in high income countries. BL in these regions is consequently referred to as “sporadic BL”. Immunodeficiency-associated Burkitt lymphoma is primarily associated with HIV infection [3]. Unlike endemic BL, sporadic and HIV-associated BL occur in all age groups. Although there are differences in clinical features and prognosis of the endemic, sporadic and HIV-associated BL [3, 4], the unifying characteristic in all patients with BL is the unique morphology and the chromosomal translocation involving MYC oncogene, which is present in BL irrespective of geographical location, and immunodeficiency status [5]. Another distinguishing feature of BL is the association with Epstein-Barr virus (EBV) infection. The endemic form is almost always EBV-positive while the sporadic BL tumours are less than 30% EBV-positive.

There have been no recent systematic attempt to estimate the actual magnitude of the burden of BL where it is known to occur at a relatively high rate, in Africa. In this report, we estimate the incidence (number of cases, and rates) of BL that occurred in Africa in 2018, and the likely fraction attributable to EB.

## Methods

### Data sources

We used the sources of information and methods employed to make national estimates of incidence for Globocan 2018 [6]. Since the subtypes of non-Hodgkin lymphoma are not reported in Globocan, we used the original sources used in the estimations, to abstract information on BL. The sources were the cancer registries of Africa, listed in Annex A of Ferlay et al. (Cancer incidence and mortality data: sources and methods by country GLOBOCAN2018_Annex_A.xlsx (available at http://gco.iarc.fr)). From these datasets, we abstracted information on cases BL (ICD-O M9687/3). In addition to these registry data, we used information on the proportions of non-Hodgkin lymphomas that were Burkitt lymphoma in unpublished registry data from Yaoundé (Cameroon)^1^ and Gabon,^2^ from newly established national paediatric registries in Burkina Faso, Republic of Congo, and Cote d’Ivoire^3^, and in published data from the Democratic Republic of Congo [7] and northern Cameroon [8].

### Method of estimation

Within the NHL catgory of Globocan 2018 (C82–86, C96 - Non-Hodgkin lymphoma) we take the proportion of BL within 5 broad age group and for each sex. These proportions were applied to the estimated number of NHL cases (by sex and age) in GLOBOCAN 2018. When the Globocan estimate derived from several cancer registries, the mean of the proportions (within age-sex groups) was used.

Incidence rates were calculated for recent periods, for males and females, for 5 broad age groups, and the age standardized incidence rates obtained (using the world standard population [9]. Registry data were available for 26 of the 48 countries of sub-Saharan Africa, and 5 of the 6 countries of Northern Africa (countries with populations < 150,000 were excluded from the analysis). For those countries for which no data were available, average incidence rates from selected neighbouring countries in the same region were used to derive national incidence within the country (*method 9*, [6]).

## Results

Table 1 shows, for the 5 regions of Africa, the estimated numbers of cases and incidence rates per 100,000 population (age specific rate in children age 0–14, and crude and age standardized rate (ASR) at all ages).

**Table 1:** Numbers and incidence rates (per 100,000 population) of Burkitt lymphoma by region and sex

REGION	MALES					FEMALES				
	Numbers		Incidence rate per 100,000			Numbers		Incidence rate per 100,000		
	0–14	TOTAL	0–14	CRUDE	ASR	0–14	TOTAL	0–14	CRUDE	ASR
Eastern Africa	1009	1191	1.09	0.51	0.38	401	487	0.44	0.22	0.19
Middle Africa	350	452	0.91	0.54	0.46	172	217	0.45	0.26	0.20
Southern Africa	13	56	0.13	0.17	0.18	5	46	0.05	0.14	0.14
Western Africa	568	719	0.67	0.37	0.29	350	451	0.43	0.24	0.21
*Sub-Saharan Africa*	*1940*	*2320*	*0.86*	*0.44*	*0.35*	*928*	*1201*	*0.42*	*0.23*	*0.20*
Northern Africa	233	292	0.59	0.24	0.23	74	87	0.20	0.07	0.07
Africa	2173	2612	0.82	0.41	0.33	1002	1288	0.39	0.20	0.17

The estimate is for a total of 3900 new cases of BL in Africa in 2018, with almost exactly two thirds in males (67%), and 81.4% of cases (3175) occurring in children age 0–14. This is almost half of all the estimated number (6474) of childhood cases of non-Hodgkin lymphoma. At all ages, incidence rates in sub-Saharan Africa do not show much variation by region (ASR’s between 0.18 and 0.46 per 100,000 in males, 0.14–0.26 per 100,000 in females), although in North Africa, the apparent rarity of BL in adult females results in a very low estimated ASR (0.07 per 100,000). In children, the highest incidence in boys is in East and Middle (Central) Africa, and in girls, the same two regions, plus West Africa. Estimated rates of childhood BL are lowest in Southern Africa.

**Fig. 1:**
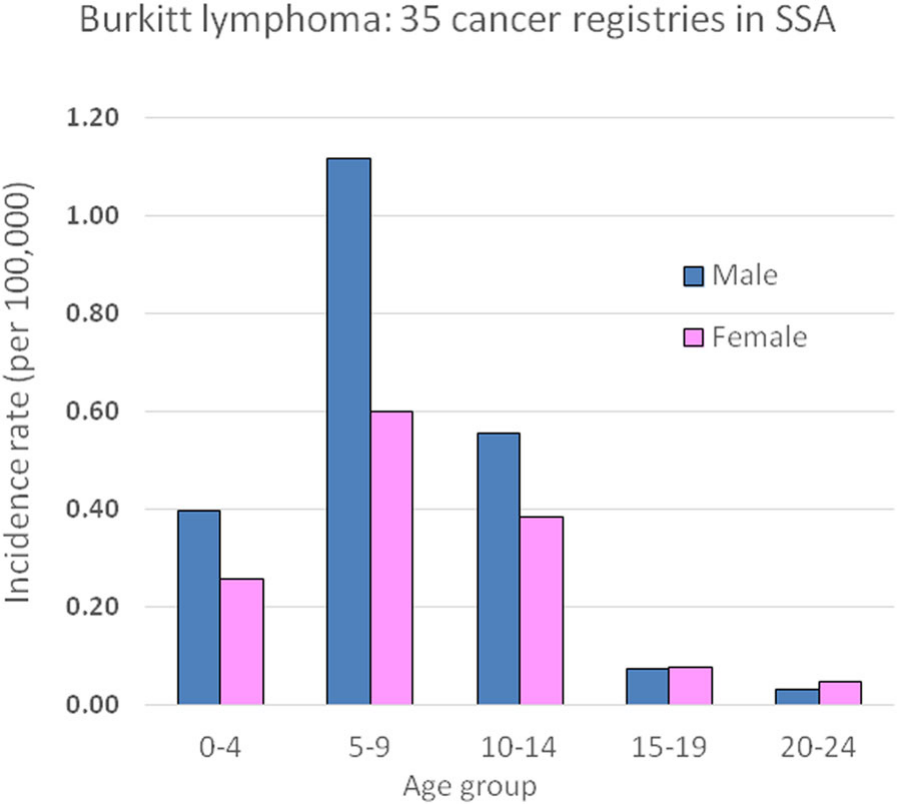
Age specific incidence rates of Burkitt lymphoma (1395 cases aged 0–24 from 35 cancer registries in sub Saharan Africa)

Figure 1 shows the incidence rates, by 5-year age group, in children and young adults (ages 0–24), pooling the data from the 35 registries contributing to the national estimates for sub Saharan Africa (857 cases in males, 538 cases in females). Incidence rates peak in the 5–9 year age group, and – in children – are higher in boys that in girls.

**Fig. 2:**
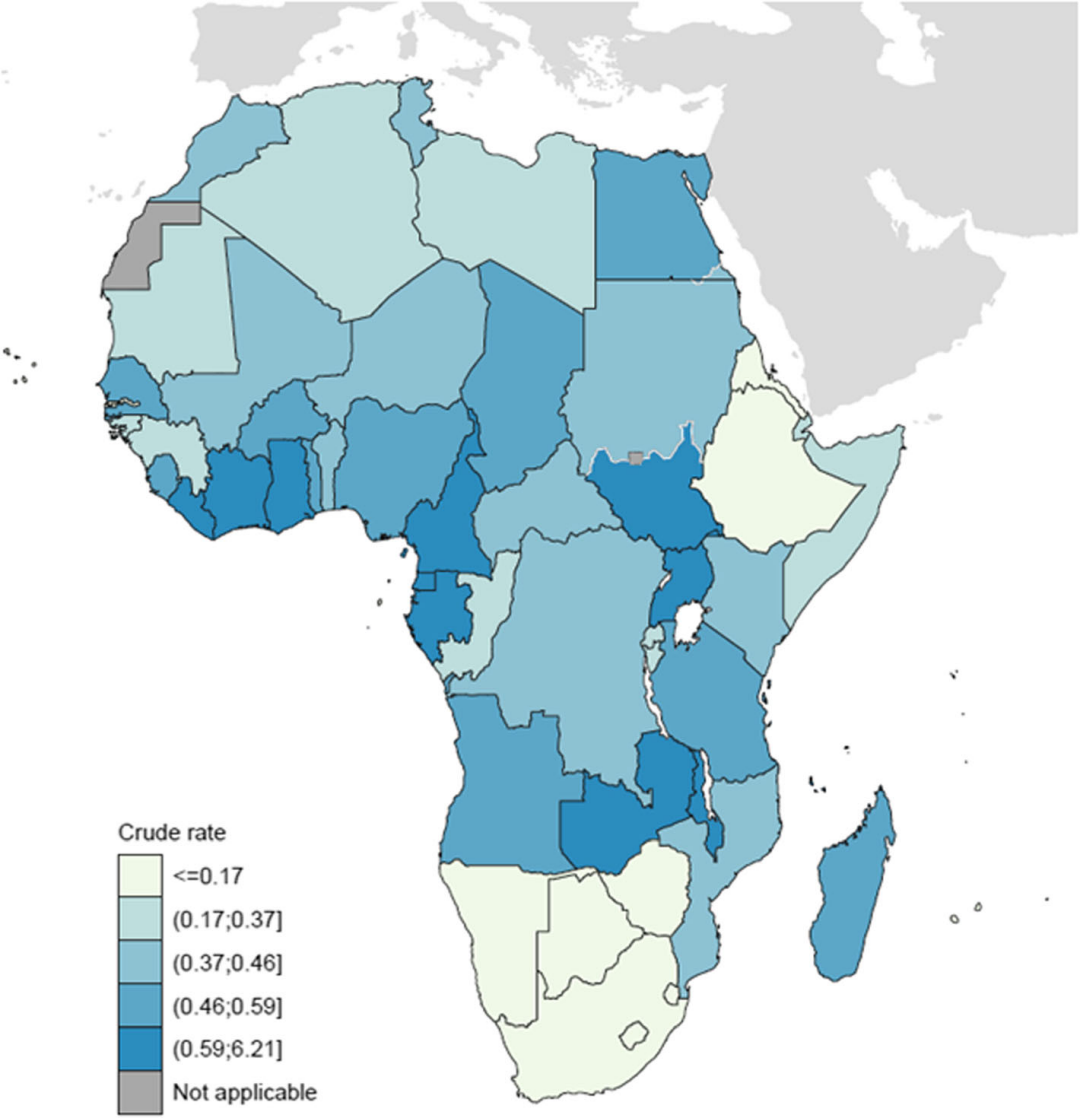
Burkitt lymphoma, patient age 0–14 years – both sexes (estimated incidence per 100,000 in 2018)

Figure 2 shows the distribution of estimated incidence rates of childhood BL at national level, as a map of Africa. The highest incidence rates are observed in Malawi (6.2 per 100,000), Cameroon (2.1), Uganda (1.4), Zambia (1.3) and Cote d’Ivoire (1.1). All of the countries of Southern Africa (as well as Ethiopia) have estimated rates of ≤0.17 per 100,000.

**Fig. 3:**
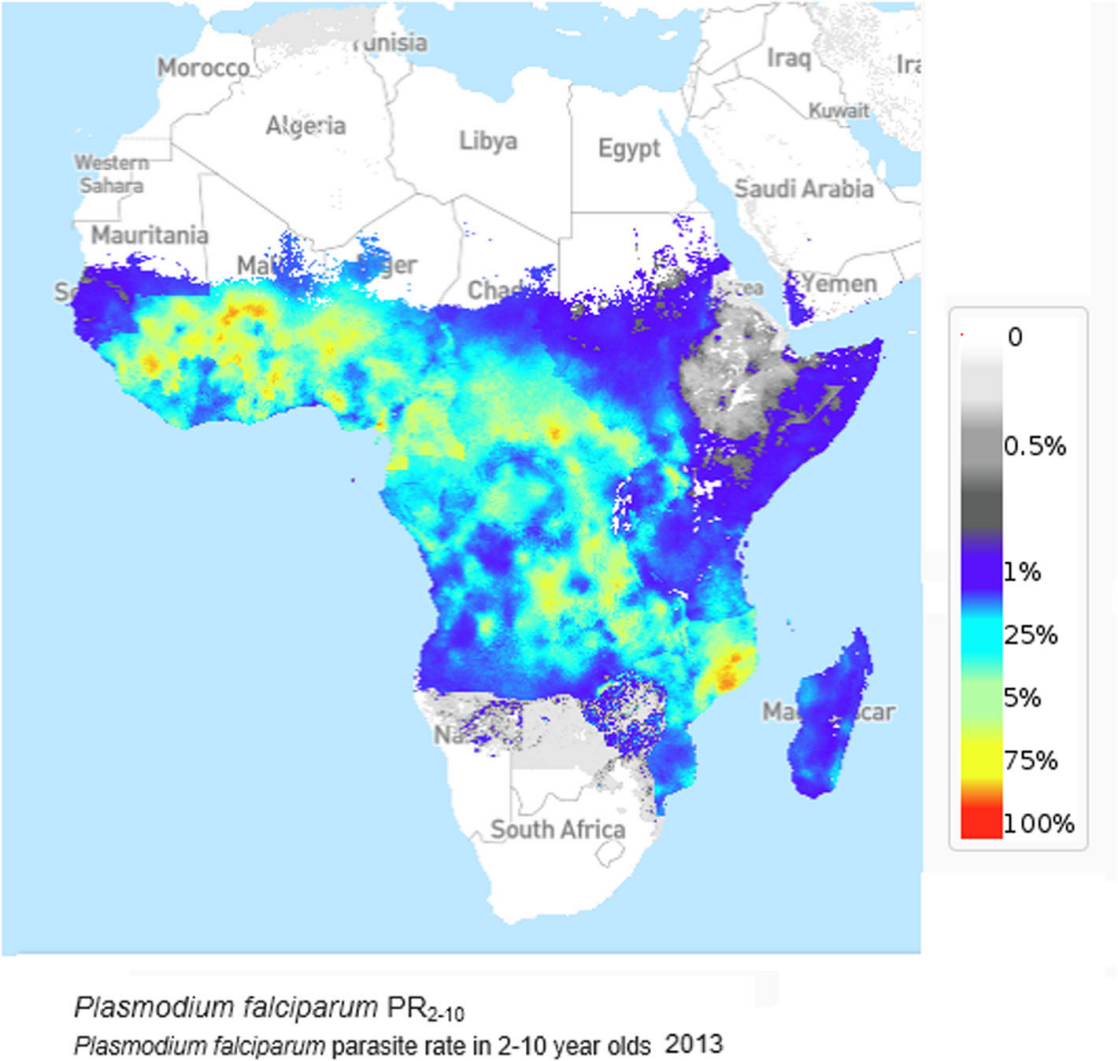
*Plasmodium falciparum* parasite rate in 2–10 year olds, Africa, 2013 [13]

In the zone of high incidence of childhood BL in central Africa, almost all cases of endemic childhood BL are associated with EBV, as demonstrated by the presence of either EBV nuclear antigen (EBNA) or EBV DNA in the tumour cells [3]. This proportion is less in cases of sporadic and immunodeficiency associated Burkitt lymphoma [10, 11]. The peculiar distribution in sub Saharan Africa is not related to EBV exposure – which is ubiquitous, but has long been linked to the geographic occurrence on malaria, particularly where it is hyper- or holo-endemic [12]. Even today, the geographic occurrence of childhood BL bears a striking resemblance to that of endemicity of malaria due to *P. falciparum* (Fig. 3).

Assuming that childhood BL in tropical Africa (Western, Middle, Eastern Africa) is of the endemic type (e-BL) with 100% EBV, while elsewhere, and among adolescents and adults, cases are of the sporadic type, with some 30% EBV associated [14], we can estimate that EBV is a causative factor in 3165 cases of BL in Africa (81% of the continental total).

## Discussion

We have used cancer registry data from Africa to derive estimates of the numbers of cases of BL occurring on the continent in 2018, using the methods developed for 36 other cancer types in Globocan 2018. Although population based cancer registration has been slowly expanding in extent and quality in recent years, with some 30 registries in sub Saharan Africa meeting criteria rendering them suitable to contribute to the national estimates of Globocan (http://afcrn.org/index.php/membership/membership-list), the data they produce are not perfect. Most score between 4 and 7 on the quality (“q”) factor used to produce uncertainty estimates in Globocan, and only 6 of the countries of Africa have registries that aim to cover the entire national population – usually only a sample of 5–10% is involved. On the positive side, BL is relatively easy to diagnose – at least in childhood - because they present as rapidly growing tumours which may affect the face with specific histological characteristics, so that case ascertainment is likely to be much better than for other tumours. Indeed, by making our estimates based on the proportions of NHL cases that are BL, we assume that relatively few of the cancers allocated to the “Lymphoma not specified” codes in ICD-O (9590/9591) will actually have been cases of BL. Most cases of BL in the registry data had been diagnosed based on histology or pathology, indeed, for most registries, all BL registrations were based on morphological verification (MV) of diagnosis. There were some important exceptions – the percentage of MV cases in the series from Ibadan (Nigeria) was 87, 71% in Blantyre (Malawi), and 65% in Kampala (Uganda).

The observations provide more extensive data to confirm earlier studies on BL incidence, indicating an excess of cases in males v females and a peak incidence of childhood BL in the 5–9 year age group. They also confirm what has long been suspected concerning the geographic distribution, and illustrates how the geography is largely mediated by the endemicity of falciparum malaria; indeed, the importance of malarial infection as a co-factor in the occurrence of BL has been demonstrated at the individual level also [15]. Previous reports have shown high rates of incidence in Malawi, Uganda, and Ibadan (Nigeria) [4, 15], Cameroon [8], northern Tanzania [16] and western Kenya [1], while the incidence rates reported from Zimbabwe and South Africa have consistently been low [17], as was the frequency of BL among NHL cases in a large clinical series (487 cases) from South Africa and Zimbabwe [18].

Our estimates of incidence are based on data actually available from Africa, almost all of which are from population based cancer registries. Most of these cover urban populations, although malaria transmission intensity is highest in rural areas. It is therefore likely that incidence of BL is higher in rural that urban areas, and there is limited data to suggest that this is so [19]. It is thus possible that the calculated numbers of BL cases in Africa are an underestimate of the true burden of BL on the continent, although they are the best that can be made with data currently available.

It is rather surprising to note that the estimated incidence of childhood is moderately elevated in some North African countries, particularly in boys. Burkitt lymphoma has reported to be a common form of childhood NHL – especially in boys - in Egypt [20] and Algeria [21], with most cases being EBV positive, and BL was found to be a moderately common form of childhood cancer in case series from Tunisia, Sudan and Morocco [22]. The absence of holoendemic malaria suggests other pathogens or environmental factors are interacting with EBV to increase the risk for BL.

A recent study by Grande et al. [23] where both EBV+ and – BL tumours were sequenced, identified EBV infection as the BL driving phenotype not the geographic origin. However, because detection of EBV in tumours is not a diagnostic criterion, we were limited in this analysis of population based cancer registries to rely on geography and age to identify the EBV+ tumours. Importantly, in the Grande study, the majority of the EBV+ tumours sequenced were from childhood BL in tropical Africa.

## Conclusions

In summary, using population based cancer registry data, we show that the burden of BL remains high in those parts of sub Saharan Africa where Falciparum malaria remains common. EBV is an etiological factor in more than 80% of cases (about 3200 in 2018).

## Data Availability

The datasets used and/or analysed during the current study are available from the AFCRN database. Requests for access should be made via the AFCRN secretariat (https://afcrn.org/index.php/research/researches-and-collaborations).

## Abbreviation

ASR: Aged Standardlised Rate
BL: Burkitt lymphoma
eBL: Endemic type BL
EBNA: EBV nuclear antigen
EBV: Epstein Barr virus
HIV: Human immunodeficiency virus
IARC: International Agency for Research on Cancer
ICD-O: International Classification of Diseases for Oncology
NHL: Non Hodgkin lymphoma
SSA: Sub-Saharan Africa

## Footnotes

1 Courtesy of Prof Georges Enow-Orok

2 Couretsy of Prof Ernest Belembaogo

3
http://afcrn.org/index.php/activities/activities-in-2018
